# Proof of principle for *piggyBac*-mediated transgenesis in the flatworm *Macrostomum lignano*

**DOI:** 10.1093/genetics/iyab076

**Published:** 2021-05-17

**Authors:** Kirill Ustyantsev, Jakub Wudarski, Igor Sukhikh, Filipa Reinoite, Stijn Mouton, Eugene Berezikov

**Affiliations:** 1 Sector of Molecular and Genetic Mechanisms of Regeneration, Institute of Cytology and Genetics SB RAS, Novosibirsk 630090, Russia; 2 Laboratory of Biological Diversity, National Institute for Basic Biology, Okazaki 444-8585, Japan; 3 European Research Institute for the Biology of Ageing, University of Groningen, University Medical Center Groningen, Groningen 9700AD, The Netherlands

**Keywords:** *piggyBac*, transposons, transgenesis, flatworms, *Macrostomum lignano*

## Abstract

Regeneration-capable flatworms are informative research models to study the mechanisms of stem cell regulation, regeneration, and tissue patterning. The free-living flatworm *Macrostomum lignano* is currently the only flatworm where stable transgenesis is available, and as such it offers a powerful experimental platform to address questions that were previously difficult to answer. The published transgenesis approach relies on random integration of DNA constructs into the genome. Despite its efficiency, there is room and need for further improvement and diversification of transgenesis methods in *M. lignano*. Transposon-mediated transgenesis is an alternative approach, enabling easy mapping of the integration sites and the possibility of insertional mutagenesis studies. Here, we report for the first time that transposon-mediated transgenesis using *piggyBac* can be performed in *M. lignano* to create stable transgenic lines with single-copy transgene insertions.

## Introduction

*Macrostomum lignano* is a free-living flatworm that is gaining attention as a powerful model organism. Thanks to its high regeneration capabilities and the availability of a robust transgenesis method, it can be used as a testbed in many research areas, including stem cell and germline biology, regeneration, and aging (Wudarski *et al.* 2020). Although the current published protocol for transgenesis by random integration of DNA constructs in *M. lignano* is easy to implement and efficient, it has several disadvantages such as high propensity to form tandem insertions, which are hard to map and can potentially affect the stability of the inserts due to recombination (Wudarski *et al.* 2017). Another drawback of the current random integration approach is the use of irradiation. Mild exposure to gamma rays causes double-strand breaks in the DNA, stimulating the repair mechanisms of the cell, and is used to increase the efficiency of integration of transgenes in the genome (Wudarski *et al.* 2017). However, the damage inflicted to the DNA can introduce alterations in the genome that are difficult to detect and correct.

DNA transposons such as *Sleeping Beauty* (Aronovich *et al.* 2011; Song *et al.* 2012), *Tol2* (Urasaki *et al.* 2008), *Mos1* (Frokjaer-Jensen *et al.* 2014), and *piggyBac* (Yusa 2015) are widely used as vectors for nonviral gene delivery in diverse animal models. Compared to random integration methods, mainly single-copy transposon insertions are easily tractable and also reversible, *i.e.*, can be removed afterward if desired (Izsvák and Ivics 2004; Frøkjær-Jensen *et al.* 2008; Lacoste *et al.* 2009). In addition, transposons offer opportunities for forward genetics studies, including insertional mutagenesis and trapping and mapping of functional DNA regulatory elements such as promoters, enhancers, and poly-adenylation signals (Bonin and Mann 2004; Kawakami *et al.* 2004; Boulin and Bessereau 2007; Rad *et al.* 2010; Song *et al.* 2012; Casandra *et al.* 2018).

In this proof of principle study, we report transposon-mediated integration of *piggyBac*-derived genetic constructs in *M. lignano* using both the original and the hyperactive versions of the *piggyBac* transposase*.* We demonstrate that this method results in stable single-copy insertions with a frequency that is acceptable for practical applications.

## Materials and methods

### *Macrostomum lignano* lines and culture conditions

The wild-type NL12 line was previously described (Wudarski *et al.* 2017). Animals were cultured in laboratory conditions in plastic Petri dishes (Greiner), filled with nutrient-enriched artificial sea water (Guillard's f/2 medium). Worms were fed ad libitum with the unicellular diatom *Nitzschia curvilineata* (Heterokontophyta, Bacillariophyceae) (SAG). Climate chamber conditions were set on 20°C with constant aeration and a 14/10 hours day/night cycle. Cultures designated for microinjection experiments were prepared as previously described (Wudarski *et al.* 2017). To speed-up the development of transgenic lines, microinjected eggs, and the subsequent progeny were kept at 25°C under otherwise the same conditions (Wudarski *et al.* 2019).

### mRNA synthesis and preparation of transgenic constructs

Codon-optimized sequences of the original (PBase) and the hyperactive (hyPBase) *piggyBac* transposases were designed using the previously established codon optimization algorithm (Wudarski *et al.* 2017) and the published sequences (Cary *et al.* 1989; Yusa *et al.* 2011). The designed sequences were commercially synthesized as gBlocks (IDT) and cloned into the pGEM-T-Easy backbone (Promega) under the *M. lignano HSP20* promoter and followed by the *M. lignano EF1a* 3' UTR. The resulting plasmids JP4 and JP5 (Supplementary Figure S1) can in principle be used to generate transgenic *M. lignano* lines with inducible transposase expression, similar to the previous heat shock inducible *M. lignano* constructs (Wudarski *et al.* 2019), but were only used in this study as PCR templates for *in vitro* synthesis of transposase mRNA. During the PCR, the T7 promoter sequence was added to the forward primer, and the product was used as a template for *in vitro* transcription. The reaction was carried out using the HiScribe T7 ARCA mRNA Kit with tailing (NEB) according to the manufacturer's instructions.

A plasmid containing *piggyBac* transposon 5' and 3' termini was made by cloning the commercially synthesized termini sequences (gBlocks, IDT) into the pGEM-T-Easy backbone (Promega). Two donor plasmid constructs were generated by cloning *(long)EF1a::mNeonGreen* (JW88), and *(short)EF1a::mNeonGreen* (KU75) fragments between the *piggyBac* termini. For the JW88 plasmid, a negative selection *DLG4::mScarlet-I* cassette was additionally cloned upstream of the transposon sequence in the *Nco*I site. See Supplementary Figure S1 for full transgene sequences and annotations.

### Microinjections, PCR screening, and insertion site identification

All microinjections were carried out following the previously published protocol (Wudarski *et al.* 2017). Only fresh, single-cell stage *M. lignano* embryos were used. Micromanipulations were done using either a microinjection stage equipped with AxioVert A1 inverted microscope (Carl Zeiss), PatchMan NP2, TransferMan NK2, FemtoJet express, and PiezoXpert (Eppendorf) or a microinjection stage equipped with PrimoVert inverted microscope (Carl Zeiss), Narishige MO-202 micromanipulators, and OpenSpritzer in-house build microinjector (Forman *et al.* 2017).

We co-injected PBase mRNA with the JW88 donor plasmid (molar ratio 10:1, final concentrations 50 and 15 ng/μl, respectively) or hyPBase mRNA with the KU75 donor plasmid (molar ratio 2:1, final concentrations 45 and 20 ng/μl, respectively) into single-cell stage *M. lignano* embryos. The resulting hatchlings were screened for the presence of fluorescent signal. In case of the KU75 plasmid, all hatchlings positive for *mNeonGreen* expression were selected. For the JW88 plasmid, which contains the additional negative selection marker *DLG4::mScarlet-I*, only *mNeonGreen*-positive/*mScarlet-I*-negative worms were selected, while double-positives animals were discarded. The selected hatchlings (P0) were paired with single wild-type NL12 worms that were raised in the same conditions. The worm pairs were transferred to fresh food every 2 weeks. For each cross, *mNeonGreen-*positive F1 animals were selected and put together on fresh food, transferred to separate dishes, and allowed to propagate. The F2 populations were selected bi-weekly and only the *mNeonGreen-*positive worms were kept. When 200 positive worms were obtained, half of each population was sacrificed for genomic DNA extraction using the QIAamp DNA Mini kit (QIAGEN) needed for subsequent PCR screens. The rest of the worms were kept to establish stable cultures.

Genomic DNA samples from the F2 *mNeonGreen-*positive worms were first screened by PCR to check for the retention of the plasmid backbone flanking the transposon cassette on both sides. In cases where no plasmid backbone was detected, we proceed to map the insertion sites.

Genomic locations and flanking sequences of the inserted *piggyBac*-derived constructs were obtained using Palindromic sequence-targeted (PST) PCR for the NL30 line and by inverse PCR for the NL31 and NL32 lines following the published protocols (Frokjaer-Jensen *et al.* 2014; Kalendar *et al.* 2019). Sanger sequencing of the gel-purified PST-PCR/inverse PCR products was done either by an external company (Eurofins, Ebersberg, Germany) or using the Big Dye Terminator V. 3.1. Cycle Sequencing Kit (Applied Biosystems). Products of sequencing reactions were analyzed using the ABI 3130xl Genetic Analyzer (Genomics Core Facility, ICBFM SB RAS, Novosibirsk, Russia), and the resulting sequences were mapped to the Mlig_3_7 *M. lignano* genome assembly (Wudarski *et al.* 2017). The insertion locations were additionally verified by PCR using primers specific for the *M. lignano* genomic DNA and the *piggyBac* transposon termini. Sequences of all primers used in the study are provided in Supplementary Table S1.

### Microscopy and imaging

Selection of positive transgenic worms and all imaging was performed using a Zeiss Axio Zoom V16 microscope with an HRm digital camera and Zeiss filter sets 38HE (FITC) and 43HE (dsRed) at the Joint Center for Microscopy of Biological Objects, of the Institute of Cytology and Genetics, SB RAS (Novosibirsk, Russia). For the imaging, worms were first starved for 48 hours, and then relaxed in 7.14% MgCl_2_ * 6H_2_O solution in Guillard's f/2 medium until they stopped moving. To bring all the organs to the focus, the relaxed worms were put in a drop of the MgCl_2_ solution on the bottom of a plastic Petri dish, and the liquid was slowly removed until the worms became gently squeezed. The images were arranged for publication using ImageJ v. 1.53c and GIMP v. 2.10.18.

### Data availability

All data generated or analyzed during this study are included in this published article and its supplementary information files. All plasmids and *M. lignano* worm lines generated in this study are available from the corresponding author on reasonable request.

Supplementary material is available at GENETICS online.

## Results and discussion

**Figure 1:**
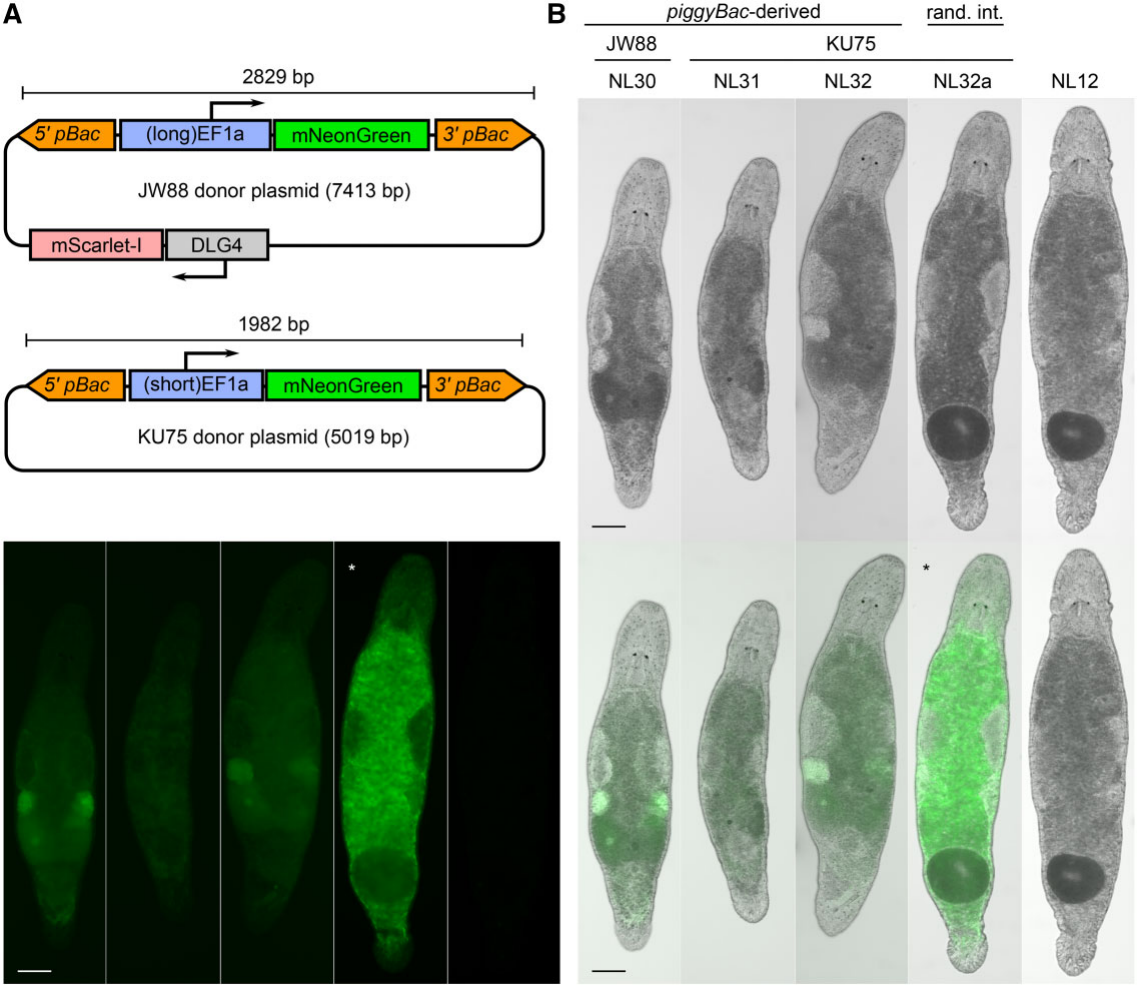
*PiggyBac*-mediated transgenesis in *M. lignano*. (A) Schematics of the *piggyBac*-derived donor plasmids JW88 and KU75 were used in this study. Blocks with thin arrows above denote the promoters with 5'UTR regions, and the directions of the arrows reflect the orientation of a gene cassette in the plasmids. 3'UTR regions are not shown. Orange arrow-shaped blocks correspond to the 5' and 3' *piggyBac* termini. Full plasmid structures and sequences can be found in Supplementary Figure S1. (B) Differences in *mNeonGreen* fluorescent signal pattern and intensity between *piggyBac*-derived transgenic lines and a random integration-derived line. Three channels are shown—brightfield, FITC (green), and their combination. Exposure time for the FITC channel was set to 100 ms in all the cases except for the random integration-derived line NL32a (25 ms, marked with an asterisk). NL12 - nontransgenic wild type line. All pictures were taken on the same day under the same magnification, differences in size are due to variation in squeeze preparations and age of the worms. Scale bar is 100 μm.

Using the microinjection approach, we delivered two sets of *piggyBac*-derived genetic constructs, JW88 and KU75, together with PBase or hyPBase mRNA into single-cell stage eggs of *M. lignano* (Table 1). Both constructs contained the sequence coding the positive selection transgenic marker *EF1a::mNeonGreen* between the *piggyBac* termini (Figure 1A). In these constructs, *mNeonGreen* is expressed under the control of a ubiquitous promoter of the *M. lignano* elongation factor alpha 1 gene (*EF1a*) with its 5'UTR, and followed by the *EF1a* 3'UTR sequence*.* The two constructs differed as follows: (1) JW88 had a longer, 1309 bp, version of the *EF1a* promoter region together with the 5'UTR, *(long)EF1a*, as originally described (Wudarski *et al.* 2017), while for the KU75 plasmid it was shortened to 465 bp, *(short)EF1a*; (2) JW88 had a negative selection cassette *DLG4::mScarlet-I* cloned outside of the transposon terminal repeats (Figure 1A). The negative selection cassette in JW88 served as a control to discriminate between actual *piggyBac*-mediated transposition and random integration in our initial experiments (Supplementary Figure S2A). The KU75 (Figure 1A, Supplementary Figure S1) construct was made as an alternative to JW88 to decrease both the cargo and overall plasmid size, which resulted in its easier propagation in *Escherichia coli*, and also lowered the extent of homology to the *M. lignano* genome, reducing potential interference.

**Table 1:** Numbers of injected eggs and progeny transmitting the *piggyBac*-derived transgenic constructs

Construct/transposase	Number of batches^*a*^	Eggs injected	Positive founders	Randomly integrated^*b*^	*piggyBac*-derived
*(long)EF1a::mNeonGreen* (JW88)/PBase	4	436	1	—	1
*(short)EF1a::mNeonGreen* (KU75)*/hyPBase*	3	393	4^*c*^	3^*c*^	2^*c*^

^*a*^ One batch corresponds to one week of injections with ∼80–150 eggs injected per week.

^*b*^ For JW88/PBase, progeny positive for *DLG4::mScarlet-I* (Supplementary Figure S2A) were not analyzed for the transgene transmission. For the KU75/hyPBase, the number is based on the PCR screening for the KU75 plasmid retention (Supplementary Figure S2B).

^*c*^ There was segregation by the transgene expression pattern and brightness in positive offspring of one of the founders.

Microinjection of 436 *M. lignano* eggs with the JW88/PBase mix over the course of 4 weeks resulted in a single green-only germline transmitting P0 worm (31 worms simultaneously positive for red and green were excluded from subsequent crossing experiments). Microinjection of 393 eggs with the KU75/hyPBase mix within 3 weeks resulted in 4 *mNeonGreen* positive transmitting founders (Table 1). Offspring of one of the KU75-based founders were visibly segregated by brightness of the green signal and were therefore split in two groups, resulting in a total of five *mNeonGreen* KU75 positive lines. Subsequent PCR screening of the F2 progeny for the retention of the plasmid sequences flanking the transposon insertions showed that two KU75-based lines had negative PCR results. The three other lines were positive (Table 1) and hence likely derived from random integrations of the KU75 plasmid (Supplementary Figure S2B).

**Figure 2:**
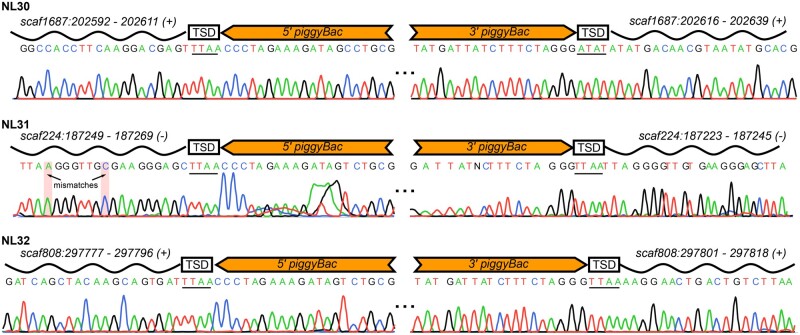
Genomic locations, flanking sequences, and TSDs of the *piggyBac*-derived transgene insertions. Partial Sanger sequence traces of PST-PCR/inverse PCR products are shown with annotations on top of the sequences. Wave-shaped lines correspond to the genomic sequences flanking the insertion sites. Mlig_3_7 genomic coordinates are given on the top of the wave-shaped lines, and orientations of the insertions are indicated in parentheses.

To map genomic sites of transgene insertions in the candidate *piggyBac*-derived lines (NL30, NL31, and NL32), we next used a combination of PST-PCR (Kalendar *et al.* 2019) and inverse PCR (Frokjaer-Jensen *et al.* 2014). We obtained the genomic sequences flanking the transgene insertions in all the transgenic lines, and mapped transgene insertion sites in the *M. lignano* genome assembly (Figure 2). In all three cases, we observed insertion patterns consistent with single-copy integration of the *piggyBac*-derived transgenes in *M. lignano* (Figure 2, Supplementary Figure S3). For the NL31 and NL32 lines (KU75 with hyPBase) the insertion site and the target site duplications (TSDs) sequences flanking the transposon insertion were the canonical TTAA (Yusa 2015), while in the line NL30 (JW88 with PBase) the insertion site was ATAT, indicating a noncanonical insertion (Figure 2). These analyses confirm that the established transgenic lines are indeed the result of *piggyBac* transposon activity. Furthermore, the inserted transgenes are stably transmitted through the germline, since their expression has remained stable for more than 10 generations for the NL30 line, and for more than 3 generations for the NL31 and NL32 lines.

Interestingly, the noncanonical TSD in the NL30 line is also asymmetric, with TTAA at the 5’ end and ATAT at the 3’ end (Figure 2). The observed TSD asymmetry can be explained if the mismatch at the 5’ end is repaired to the canonical *piggyBac* TTAA site while the 3’ end mismatch is repaired to the host genome variant ATAT (Supplementary Figure S4). Whether this is a frequent or rare integration site in the case of *piggyBac* activity in *M. lignano* is still to be determined. Previous research in human embryonic stem cells showed that noncanonical insertions of *piggyBac* can happen in 2% of the integration cases, and that the mismatch in the sequence is repaired by the repair mechanism of the host cell (Li *et al.* 2013). Deviation from the canonical insertion pattern might indicate that some *M. lignano* cellular factors can somehow intervene with the transposition process through interaction with the transposase/transposon nucleoprotein complexes (Feschotte 2006; Kolacsek *et al.* 2014).

All three *piggyBac*-derived lines express *mNeonGreen* at visibly lower levels than the NL31a line resulted from random integration, which corresponds to the expected low number of transgene copies integrated by transposition. Importantly, there was no significant difference in either brightness or expression pattern between the NL30 and NL32 lines, which are derived from different donor constructs (Figure 1B). This suggests that the shorter version of the *EF1a* promoter has all required regulatory elements and can thus be used instead of the longer version. The third *piggyBac*-based line, NL31, showed an overall lower expression level of *mNeonGreen* and lacks expression in the ovaries and developing eggs when compared to NL32, which is based on the same KU75 construct (Figure 1B). This difference in the expression patterns is most likely explained by the insertion position effect and emphasizes the need of generating multiple transgenic lines when investigating expression patterns of different promoters.

Here, we showed that both variants of the codon-optimized *piggyBac* transposases PBase (the original insect sequence) and hyPBase (the artificial variant with 7 amino acid mutations) (Cary *et al.* 1989; Yusa *et al.* 2011) are active in *M. lignano* (Figure 1). Based on the previous studies in mammals, hyPBase should have demonstrated several fold higher excision and integration efficiencies compared to PBase (Yusa *et al.* 2011; Burnight *et al.* 2012). However, we did not observe significantly higher number of *piggyBac*-derived transgenic *M. lignano* worms with hyPBase compared to PBase (Table 1). Therefore, we cannot conclude that hyPBase has higher efficiency compared to PBase in our setting, although optimization of transposase to transposon ratio might be the issue (Wu *et al.* 2006) and will be the subject of further optimization.

Although using a plasmid construct with a negative selection marker (like JW88) can potentially save a lot of time on the subsequent screening of transposon-derived insertions, the approach has several caveats. Apart from the more difficult propagation of plasmids of larger size in *E. coli* and the potential interference between regulatory elements, there is no guarantee that the plasmid will not be linearized somewhere in the negative selection marker sequence, which would lead to the absence of its expression and, thus, to false-positive conclusion that the event was *piggyBac*-derived. It also appears that it is possible to distinguish a high-copy random integration event from a single-copy transposon insertion by eye, as the latter appears evidently dimmer (Figure 1B). Therefore, shorter donor vectors like KU75 may be more beneficial for future applications of *piggyBac*-based transgenesis in *M. lignano*.

## Funding

The work of Kirill Ustyantsev and Eugene Berezikov on construction of the KU75-based *piggyBac*-derived transgenic constructs, generation, and the analysis of transgenic worms was supported by the Russian Science Foundation grant № 20-14-00147 and performed in Institute of Cytology and Genetics SB RAS. General maintenance of worm cultures was performed in Institute of Cytology and Genetics SB RAS by Igor Sukhikh and supported by the State budget project 0259-2021-0013. The work on generating JW88-based transgenic animals was performed at the European Institute for the Biology of Ageing and supported by the Dutch Research Council grant OCENW.KLEIN.054 to Eugene Berezikov. Filipa Reinoite was supported by the University of Groningen Graduate School of Medical Sciences Fellowship.

## Conflicts of interest

None declared.

## Supplementary Material

iyab076_Supplementary_Data
