# Erratum to: Proof of principle for *piggyBac*-mediated transgenesis in the flatworm *Macrostomum lignano*

**DOI:** 10.1093/genetics/iyab219

**Published:** 2021-12-30

**Authors:** Kirill Ustyantsev, Jakub Wudarski, Igor Sukhikh, Filipa Reinoite, Stijn Mouton, Eugene Berezikov

*Genetics*, 2021, https://doi.org/10.1093/genetics/iyab076

In the originally published version of this manuscript, there was an inaccuracy in a reference in the PDF to the Supplementary material for the article and the Supplementary material was omitted from the online version of the paper. The reference should read: “Supplementary Material is available at *GENETICS* online.” instead of “Supplementary material is available at figshare DOI: https://doi.org/10.25386/genetics.14558805.” The reference error has now been corrected and the supplementary material supplied online.

