# Supplementary material for: Proof of principle for piggyBac-mediated transgenesis in the flatworm Macrostomum lignano
Source: Genetics. 2021 May 17;218(3):iyab076. doi: 10.1093/genetics/iyab076 (PMC8717057; doi:10.1093/genetics/iyab076)
Supplement: iyab076_Supplementary_Data [file iyab076_supplementary_data.zip › iyab076/GENETICS-2021-304273_Figure_S1.pdf]

Figure S1. *PiggyBac*-derived plasmids maps, primers, promoters and codon-optimized sequences used for creating transgenic constructs.

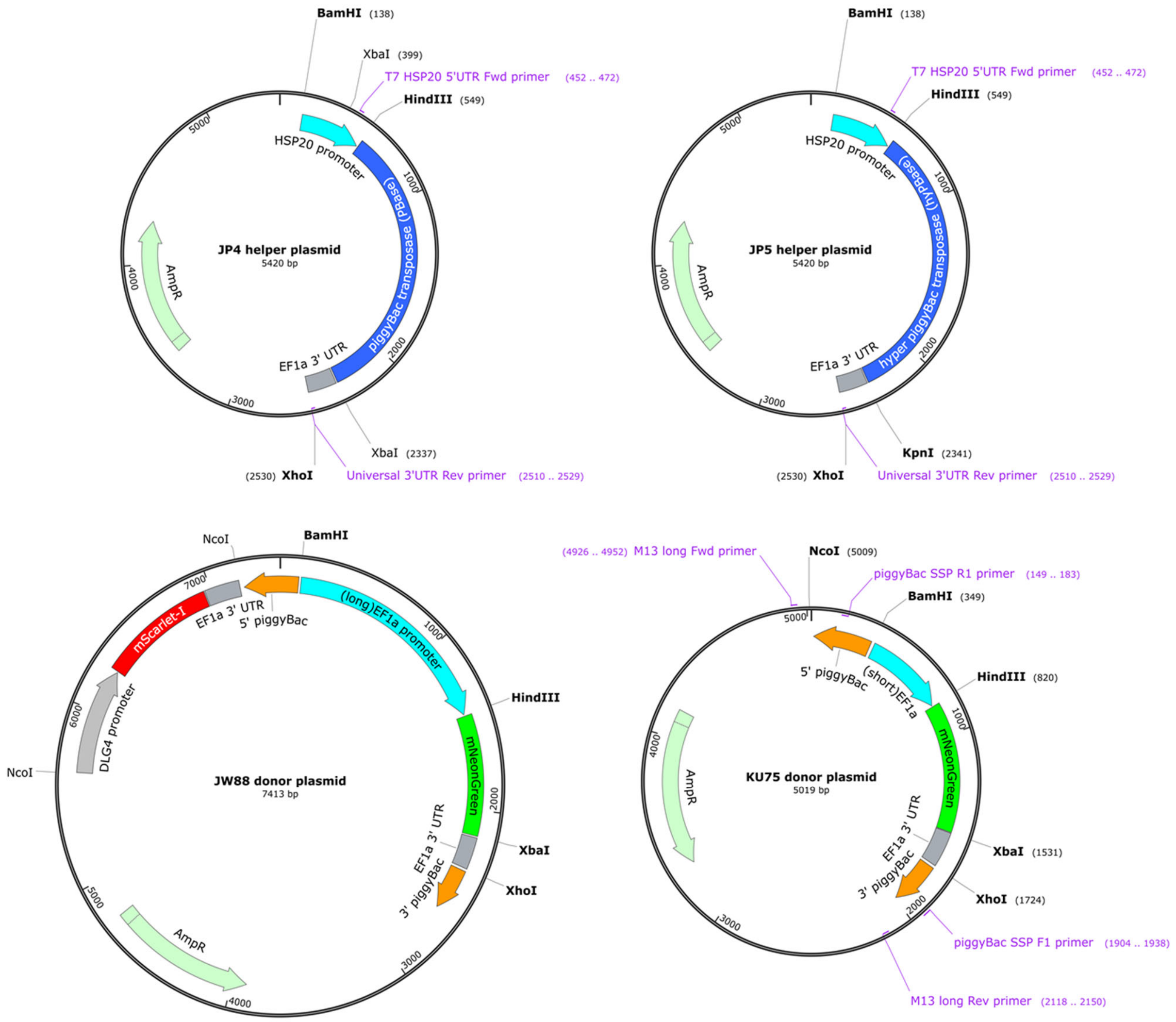

**Annotation**	**Sequence (5' – 3')**
T7 HSP20 Fwd primer	*TAATACGACTCACTATAGGG* AGTCTATTTAAGCTAGAAAGC
Universal 3'UTR Rev primer	AGTCAGGAAATACTAGCAAC
M13 long Fwd primer	CGACGTTGTAAAACGACGGCCAGTGAA
M13 long Rev primer	ACAGGAAACAGCTATGACCATGATTACGCCAAG
piggyBac SSP F1 primer	ACCTCGATATACAGACCGATAAAACACATGCGTCA
piggyBac SSP R1 primer	TTCAAAATCAGTGACACTTACCGCATTGACAAGCA
HSP20 promoter (done by genomic DNA	**[BamHI]**<br>TCATTTATAAGCGTACCGTACTTTTAAATCGTTGTTGATCTCATTATTTTAATATCTGATTTAATAATTTAGAAAAAAAA<br>ATTGATTTAACTGATTAACTTATCAGTTATAATTATAATGAAAACAAATCTAGTCTTTCTTGAGAAAATATAAGCATTCT

PCR), JP4 and JP5 plasmids	AAAATATGTACGACATTTTGCTAATTTTTCTACTGTTAGTAGCTAAAGTAGCCCTCACAAGATTAATATGTTGAGACACG<br>TCAACTTAAGAACATTCTAGAATTTTCTTAACGCATTACAATTTGGAATATTCTGGAAGGTCAGAGCAAGTCTATTTAAG<br>CTAGAAAGCACATCGTGTTTAGAAGTTAAGTTTTCACCGAACTGTTCAAATCA<br>GCAATTATAATACGCCAGTCAACAACAGC**ATG**<br>**[HindIII]**
(long)EF1a promoter (done by genomic DNA PCR), JW88 plasmid	**[BamHI]**<br>CCTATCCTGAGTCCAGTTTTCATCGCTTTTTGCCAAATCCAGCTTCCTACTAGTGTCATGGTGAGCAGAATGACATCATT<br>TGTGTACATAGGCTACACGGGCTCCACTAGCCTAGCTTTGAGTTTAGTTTGATCCGGCCGTTTGATTCTTTTTGTACATT<br>GGTCGTGTAATTTAGTGGCTCATTTGATGAGTGTGAATGAGCCTTAATGTGTAAATTAAATGCAATAAATGCACTATATT<br>TTAAATTGTTTATATGTAGGATCTTTGGCAGTCCAGTAGTATAGTGTTCGTCTACCAGCTTAAGCCAAACATTTAGAGAT<br>AATTCATGCAAGCCTGCAAAAGCTGAAAACAAAGTAAAGAGATTTTTCATTGTTTCTTTAGCAATTAAAAATTATTAATT<br>CGATTTCATACAACACATATGCAGTCAAAATTCAGACCTCTAAGCTGGTACCAATCATTTTTTTCCTATCATGATGTACG<br>TGTACGGGTTGCTTTGTTTTTTATGTAATATTTGCTGTGTTTTACTGTTGTTTTAGCTTATTTACTTCTACATGCAGCAA<br>AATCCTATACAAATCCTAAAATCACAATATACTAAATCTCTAAGGTTTGACCGGTATTCCAGTCTTTAAGTGTACTAAAG<br>GTGATACCAGGGATGGACATTCTAGCAGTGCAGGCTATTATAAGGTTTTTGGCCTGGCCTATGCATTAAATATTTTTATT<br>GTACACTTAAAAACTAGTAACAGAGTGAATTATTGCACATTCAAATTTATAAAAATTTGTAATTTTTTTCATTTAATTGT<br>CATTAAAAATATTTCATCCGTCAAGCCGTCCACATAAAATTAGTATACGTCTTCTGTTCAGTGTTACAAGTTTTGATGTA<br>AAAAACAATATTGGCCTATAACATTTTCATATATCCTCTCTACATTTTCATATATTTTAAAAAGTATTTAAAAAACATCT<br>TAAAAGTATTCCATTTTATTATTAACTGAAAAACTAACGTAAAATTACAATATATCTCAAAACTTTCTAAAGTCGTATAT<br>TTTCCGGCATCGTTAATTTTAACGACAATCGTGAAAGTTCGTGAACAAGTTTGCTGTTCCTCACCTAAATTTGAATTACT<br>ATCGCTCTTGTCTGAATGGATTTCTTACTGCAGATATCCTGAAGTAAGTCTTTCAATTTGTGAATTGTAAGTAAAGTATT<br>TTTTATTACTGCTATCATATTTGTTGAATTTGTTCCAAATTACTATTTCTTGCTTTTAATTCAGTTCCTTTTATTTATTC<br>CTAAACTCAGGTAGTTTAACAACAGCATT**ATG[HindIII]**
(short)EF1a promoter (done by genomic DNA PCR), KU75 plasmid	**[BamHI]**<br>CGTCTTCTGTTCAGTGTTACAAGTTTTGATGTAAAAAACAATATTGGCCTATAACATTTTCATATATCCTCTCTACATTT<br>TCATATATTTTAAAAAGTATTTAAAAAACATCTTAAAAGTATTCCATTTTATTATTAACTGAAAAACTAACGTAAAATTA<br>CAATATATCTCAAAACTTTCTAAAGTCGTATATTTTCCGGCATCGTTAATTTTAACGACAATCGTGAAAGTTCGTGAACA<br>AGTTTGCTGTTCCTCACCTAAATTTGAATTACTATCGCTCTTGTCTGAATGGATTTCTTACTGCAGATATCCTGAAGTAA<br>GTCTTTCAATTTGTGAATTGTAAGTAAAGTATTTTTTATTACTGCTATCATATTTGTTGAATTTGTTCCAAATTACTATT<br>TCTTGCTTTTAATTCAGTTCCTTTTATTTATTCCTAAACTCAGGTAGTTTAACAACAGCATT**ATG**<br>**[HindIII]**
DLG4 promoter (done by genomic DNA PCR), JW88 plasmid	**[NcoI]**<br>TCCGAAAATAAGCCTAATCAGACAAACCCCCAAGTGCAATATACAAATTAATCAATAAAATTTTGTGCCAGAAAACTGCA<br>AAGTAAATATATAGAAAATGCCAGCTGATTATTTCAGTACGAAAATAAATAAATTAGTTAGCGCACAATTTGACATAGCT<br>ACATTTTGCGGATATTTATTATGCACAATATGCCGATAAAATAGTAAACTATAGAAGGTACCTGGAGGTTTGGTGTCTAA<br>GCGTCTTCTTGTTGAAATTGCGAAGTGATTTCGCAGAAAAAGCTATTTTTAAGTTAGCAGGTGTTCGTTTTAACAAATCA<br>GCTTTTAGCTTTGTACTTCTATTTAAATTTATCTTCTAAACTGTTAAAAAACTCTTTTAAAAAGAATTTTAAGTTATTTT<br>CAATTTAATATTGTGCTGAATTCACTAAAATACGTTTTAAGTATATGCATTTCCTTTTGCTTTTTGAAGCTGGTCTTTTC<br>CGCATTACTAAAAGCTAAGTTCAAAGACTAGGCCTGTGATATCTGAGCCACTGTCATAAAAAACAATAGAAATATTCTCC<br>TTTATTTAGGTTGAGTTGTCTCTAAATTGATTATTTACTCCTCTAAAATTATTATTAGAATAGGGA**ATG**<br>**[NheI]**
piggyBac transposase, codon optimized, PBase, done by GeneBlock, JP4 plasmid	**[HindIII]**<br>ATCGGCAGCAGCCTGGACGACGAGCACATCCTGAGCGCCCTGCTGCAAAGCGACGACGAGCTGGTGGGCGAGGACTCCGA<br>TTCCGAGATCAGCGACCACGTGAGCGAGGACGACGTGCAGAGCGACACCGAGGAAGCCTTCATCGACGAGGTGCACGAGG<br>TGCAGCCGACCAGCAGCGGCAGCGAGATCCTGGACGAGCAGAACGTGATCGAGCAGCCGGGCAGCAGCCTGGCCAGCAAC<br>CGCATCCTGACCCTGCCGCAGCGCACCATCCGCGGCAAGAACAAGCACTGCTGGAGCACCAGCAAGAGCACCCGCCGCAG<br>CCGCGTGAGCGCCCTGAACATCGTGCGCAGCCAGCGCGGCCCGACCCGCATGTGCCGCAACATCTACGACCCGCTGCTGT<br>GCTTCAAGCTGTTCTTCACCGACGAGATCATCAGCGAGATCGTGAAGTGGACCAACGCCGAGATCAGCCTGAAGCGCCGC<br>GAGAGCATGACCGGCGCCACCTTCCGCGACACCAACGAGGACGAGATCTACGCCTTCTTCGGCATCCTGGTGATGACCGC<br>CGTGCGCAAGGACAACCACATGAGCACCGACGACCTGTTCGACCGCAGCCTGAGCATGGTGTACGTGAGCGTGATGAGCC<br>GCGACCGCTTCGACTTCCTGATCCGCTGCCTGCGCATGGACGACAAGAGCATCCGCCCGACCCTGCGCGAGAACGACGTG<br>TTCACCCCGGTGCGCAAGATCTGGGACCTGTTCATCCACCAGTGCATCCAGAACTACACTCCGGGCGCCCACCTGACCAT<br>CGACGAGCAGCTGCTGGGCTTCCGCGGCCGGTGCCCGTTCCGCATGTACATCCCGAACAAGCCGAGCAAGTACGGCATCA<br>AGATCCTGATGATGTGCGACAGCGGCACCAAGTACATGATTAACGGTATGCCGTACCTGGGCCGCGGCACCCAGACCAAC<br>GGCGTGCCGCTGGGCGAGTACTACGTGAAGGAGCTGTCCAAGCCGGTGCACGGCTCCTGCCGCAACATCACTTGCGACAA<br>TTGGTTCACCAGCATTCCGCTGGCCAAGAACCTGCTCCAGGAGCCGTACAAGCTGACCATCGTGGGCACCGTGCGCAGCA<br>ACAAGCGCGAGATCCCGGAGGTGCTGAAGAACAGCCGCAGCCGCCCGGTGGGCACCAGCATGTTCTGCTTCGACGGCCCG<br>CTGACCCTGGTGAGCTACAAGCCGAAGCCGGCCAAGATGGTGTACCTGCTGAGCAGCTGCGACGAGGACGCCAGCATCAA<br>CGAGAGCACCGGCAAGCCGCAGATGGTGATGTACTACAACCAGACCAAGGGCGGCGTGGACACCCTGGACCAGATGTGCA<br>GCGTGATGACTTGCAGCCGCAAGACCAACCGCTGGCCGATGGCCCTGCTGTACGGCATGATTAACATCGCCTGCATCAAC<br>AGCTTCATCATCTACAGCCACAACGTGAGCAGCAAGGGCGAGAAGGTGCAGAGCCGCAAGAAGTTCATGCGCAACCTGTA<br>CATGAGCCTGACCAGCAGCTTCATGCGCAAGCGCCTGGAGGCCCCGACCCTGAAGCGCTACCTGCGCGACAACATCAGCA<br>ACATCCTGCCGAACGAGGTGCCGGGCACCAGCGACGACAGCACCGAGGAGCCGGTGATGAAGAAGCGCACCTACTGCACC<br>TACTGCCCGAGCAAGATCCGCCGCAAGGCCAACGCCAGCTGCAAGAAGTGCAAGAAGGTGATCTGCCGCGAGCACAACAT<br>CGACATGTGCCAGAGCTGCTTC<br>**[XbaI]**
hyperactive *piggyBac* transposase, hyPBase, codon optimized, done by GeneBlock, JP5 plasmid	**[HindIII]**<br>GGCAGCAGCCTGGACGACGAGCACATCCTGAGCGCCCTGCTGCAGAGCGACGACGAGCTGGTGGGCGAGGACAGCGACAG<br>CGAGGTGAGCGACCACGTGAGCGAGGACGACGTGCAGAGCGACACCGAGGAGGCCTTCATCGACGAGGTGCACGAGGTGC<br>AGCCGACCAGCAGCGGCAGCGAGATCCTGGACGAGCAGAACGTGATCGAGCAGCCGGGCAGCAGCCTGGCCAGCAACCGC<br>ATCCTGACCCTGCCGCAGCGCACCATCCGCGGCAAGAACAAGCACTGCTGGTCCACCAGCAAGCCGACCCGCCGCAGCCG<br>CGTGAGCGCCCTGAACATCGTGCGCAGCCAGCGCGGCCCGACCCGCATGTGCCGCAACATCTACGACCCGCTGCTGTGCT<br>TCAAGCTGTTCTTCACCGACGAGATCATCAGCGAGATCGTGAAGTGGACCAACGCCGAGATCAGCCTGAAGCGCCGCGAG<br>AGCATGACCAGCGCCACCTTCCGCGACACCAACGAGGACGAGATCTACGCCTTCTTCGGCATCCTGGTGATGACCGCCGT<br>GCGCAAGGACAACCACATGAGCACCGACGACCTGTTCGACCGCAGCCTGAGCATGGTGTACGTGAGCGTGATGAGCCGCG<br>ACCGCTTCGACTTCCTGATCCGCTGCCTGCGCATGGACGACAAGAGCATCCGCCCGACCCTGCGCGAGAACGACGTGTTC

	ACCCCGGTGCGCAAGATCTGGGACCTGTTCATCCACCAGTGCATCCAGAACTACACCCCGGGCGCCCACCTGACCATCGA<br>CGAGCAGCTGCTGGGCTTCCGCGGCCGCTGCCCGTTCCGCGTGTACATCCCGAACAAGCCGAGCAAGTACGGCATCAAGA<br>TCCTGATGATGTGCGACAGCGGCACCAAGTACATGATCAACGGCATGCCGTACCTGGGCCGCGGCACCCAGACCAACGGC<br>GTGCCGCTGGGCGAGTACTACGTGAAGGAGCTGAGCAAGCCGGTGCACGGCAGCTGCCGCAACATCACCTGCGACAACTG<br>GTTCACCAGCATCCCGCTGGCCAAGAACCTGCTGCAGGAGCCGTACAAGCTGACCATCGTGGGCACCGTGCGCAGCAACA<br>AGCGCGAGATCCCGGAGGTGCTGAAGAACAGCCGCAGCCGCCCGGTGGGCACCAGCATGTTCTGCTTCGACGGCCCGCTG<br>ACCCTGGTGAGCTACAAGCCGAAGCCGGCCAAGATGGTGTACCTGCTGAGCAGCTGCGACGAGGACGCCAGCATCAACGA<br>GAGCACCGGCAAGCCGCAGATGGTGATGTACTACAACCAGACCAAGGGCGGCGTGGACACCCTGGACCAGATGTGCAGCG<br>TGATGACCTGCAGCCGCAAGACCAACCGCTGGCCGATGGCCCTGCTGTACGGCATGATCAACATCGCCTGCATCAACAGC<br>TTCATCATCTACAGCCACAACGTGAGCAGCAAGGGCGAGAAGGTGCAGAGCCGCAAGAAGTTCATGCGCAACCTGTACAT<br>GGGCCTGACCAGCAGCTTCATGCGCAAGCGCCTGGAGGCCCCGACCCTGAAGCGCTACCTGCGCGACAACATCAGCAACA<br>TCCTGCCGAAGGAGGTGCCGGGCACCAGCGACGACAGCACCGAGGAGCCGGTGATGAAGAAGCGCACCTACTGCACCTAC<br>TGCCCGAGCAAGATCCGCCGCAAGGCCAGCGCCAGCTGCAAGAAGTGCAAGAAGGTGATCTGCCGCGAGCACAACATCGA<br>CATGTGCCAGAGCTGCTTCTAA<br>**[KpnI]**
5' piggyBac-><br><- 3' piggyBac, done by GeneBlock., JW88 and KU75 plasmids	TTAACCCTAGAAAGATAGTCTGCGTAAAATTGACGCATGCATTCTTGAAATATTGCTCTCTCTTTCTAAATAGCGCGAAT<br>CCGTCGCTGTGCATTTAGGACATCTCAGTCGCCGCTTGGAGCTCCCGTGAGGCGTGCTTGTCAATGCGGTAAGTGTCACT<br>GATTTTGAACTATAACGACCGCGTGAGTCAAAATGACGCATGATTATCTTTTACGTGACTTTTAAGATTTAACTCATACG<br>ATAATTATATTGTTATTTCATGTTCTACTTACGTGATAACTTATTATATATATATTTTCTTGTTATAGATATCAACTAGA<br>ATGCTAGCATGGGC **[BamHI] [XhoI]** ATAATCGAATTCATGTCGACATACTAGTTAAAAGTTTTGTTACTTTATAG<br>AAGAAATTTTGAGTTTTTGTTTTTTTTTAATAAATAAATAAACATAAATAAATTGTTTGTTGAATTTATTATTAGTATGT<br>AAGTGTAAATATAATAAAACTTAATATCTATTCAAATTAATAAATAAACCTCGATATACAGACCGATAAAACACATGCGT<br>CAATTTTACGCATGATTATCTTTAACGTACGTCACAATATGATTATCTTTCTAGGGTTAA
mNeonGreen, codon optimized, done by GeneBlock, JW88 and KU75 plasmids	**[HindIII]**<br>GTGAGCAAGGGCGAGGAGGACAACATGGCCAGCCTGCCGGCCACCCACGAGCTGCACATCTTCGGCAGCATCAACGGCGT<br>GGACTTCGACATGGTGGGCCAGGGCACCGGCAACCCGAACGACGGCTACGAGGAGCTGAACCTGAAGAGCACCAAGGGCG<br>ACCTtCAGTTCAGCCCGTGGATtCTGGTGCCGCACATCGGCTACGGCTTCCACCAGTACCTGCCGTACCCGGACGGCATG<br>AGCCCGTTCCAGGCCGCtATGGTGGACGGCAGCGGCTACCAGGTGCACCGCACCATGCAGTTCGAGGACGGCGCCAGCCT<br>GACCGTGAACTACCGCTACACCTACGAGGGCAGCCACATCAAGGGCGAGGCCCAGGTGAAGGGCACCGGCTTCCCGGCCG<br>ACGGCCCGGTGATGACCAACAGCCTGACCGCCGCCGACTGGTGCCGCAGCAAGAAGACCTACCCGAACGACAAGACCATC<br>ATCAGCACCTTCAAGTGGAGCTACACCACCGGCAACGGCAAGCGCTACCGCAGCACCGCCCGCACCACCTACACCTTCGC<br>CAAGCCGATGGCCGCCAACTACCTGAAGAACCAGCCGATGTACGTGTTCCGCAAGACCGAGCTGAAGCACAGCAAGACCG<br>AGCTGAACTTCAAGGAGTGGCAGAAGGCCTTCACCGACGTGATGGGCATGGACGAGCTGTACAAG<br>**[XbaI]**
mScarlet-I, codon optimized, done by GeneBlock, JW88 plasmid	**[NheI]**<br>GTGAGCAAGGGCGAGGCCGTGATCAAGGAGTTCATGCGCTTCAAGGTGCACATGGAGGGCAGCATGAACGGCCACGAGTT<br>CGAGATCGAGGGCGAGGGCGAGGGCCGCCCGTACGAGGGCACCCAGACCGCCAAGCTGAAGGTGACCAAGGGCGGCCCGC<br>TGCCGTTCAGCTGGGACATCCTGAGCCCGCAGTTCATGTACGGCAGCCGCGCCTTCATCAAGCACCCGGCCGACATCCCG<br>GACTACTACAAGCAGAGCTTCCCGGAGGGCTTCAAGTGGGAGCGCGTGATGAACTTCGAGGACGGCGGCGCCGTGACCGT<br>GACCCAGGACACCAGCCTGGAGGACGGCACCCTGATCTACAAGGTGAAGCTGCGCGGCACCAACTTCCCGCCGGACGGCC<br>CGGTGATGCAGAAGAAGACtATGGGCTGGGAGGCCAGCACCGAGCGCCTGTACCCGGAGGACGGCGTGCTGAAGGGCGAC<br>ATCAAGATGGCCCTGCGCCTGAAGGACGGCGGtCGCTACCTGGCCGACTTCAAGACCACCTACAAGGCCAAGAAGCCGGT<br>GCAGATGCCGGGCGCCTACAACGTGGACCGCAAGCTGGACATCACCAGCCACAACGAGGACTACACCGTGGTGGAGCAGT<br>ACGAGCGCAGCGAGGGCCGCCACAGCACCGGCGGCATGGACGAGCTGTACAAG<br>**[KpnI]**
3'UTR (done by genomic DNA PCR), all plasmids	**[KpnI/XbaI]**<br>**TGA**AATGACCCGCACCTGTGTAATTCTGGAGGCATTGCAGTTAACTGAACTATTTGGAATGCTCGATTGTCGATCAATAA<br>AGAATGCGAAGTTAAAGTTGCGAGACTTGTTTGTTTGGAAATTCCTGTCTCGATTTGTTTAATTTCTTTGTGTTTCAATC<br>TATTTGTTGCTAGTATTTCCTGACT<br>**[XhoI]**
