# Supplementary material for: Proof of principle for piggyBac-mediated transgenesis in the flatworm Macrostomum lignano
Source: Genetics. 2021 May 17;218(3):iyab076. doi: 10.1093/genetics/iyab076 (PMC8717057; doi:10.1093/genetics/iyab076)
Supplement: iyab076_Supplementary_Data [file iyab076_supplementary_data.zip › iyab076/GENETICS-2021-304273_Figure_S3.pdf]

A

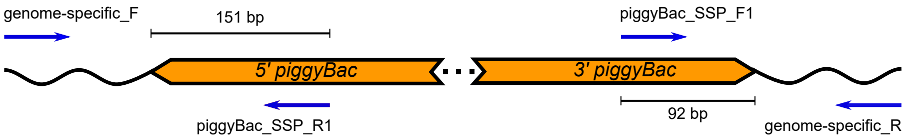

Expected 5' PCR product length (bp)	*M. lignano* line	Expected 3' PCR product length (bp)
190	NL30	289
2381	NL31	621
647	NL32	569
-	NL12	-

B

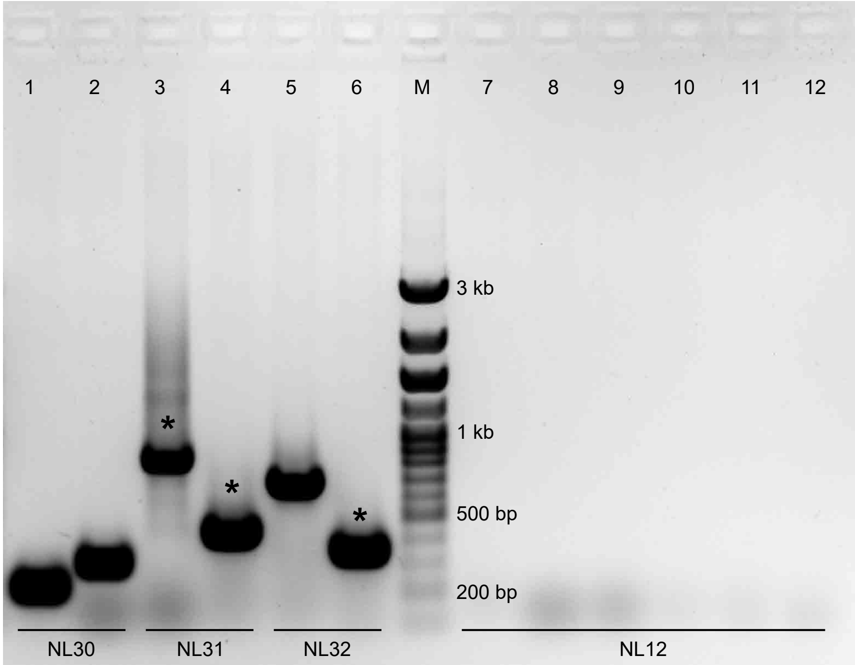

Figure S3. Validation of *piggyBac*-derived transgene insertions by PCR using *M. lignano* genomic DNA specific primers. NL30-NL32 - *piggyBac*-derived transgenic lines obtained in this study. NL12 - non-transgenic wild type line. Names and sequences of the used primer pairs are in Table S1. (A) Schematics of the PCR reactions and expected length of the PCR products corresponding to mapped *piggyBac* locations as in Figure 2. (B) Separation of PCR products in a 1% agarose gel stained with EtBr is shown. Primers used for the PCR reaction in the lanes 1–6 were the same for the lanes 7–12. Lane M - marker DNA (Step 100 Long, Biolabmix, Novosibirsk); Lanes 1, 3, and 5 - mapping of the 5' piggyBac terminus. Lanes 2, 4, and 6 - mapping of the 3' piggyBac terminus. Sizes of the bands marked with asterisks do not exactly correspond to the expected values due to extended tandem repeat areas flanking the transposon insertion sites, which might be incorrectly assembled in the Mlig_3_7 genome assembly.
