# Supplementary material for: Proof of principle for piggyBac-mediated transgenesis in the flatworm Macrostomum lignano
Source: Genetics. 2021 May 17;218(3):iyab076. doi: 10.1093/genetics/iyab076 (PMC8717057; doi:10.1093/genetics/iyab076)
Supplement: iyab076_Supplementary_Data [file iyab076_supplementary_data.zip › iyab076/GENETICS-2021-304273_Figure_S2.pdf]

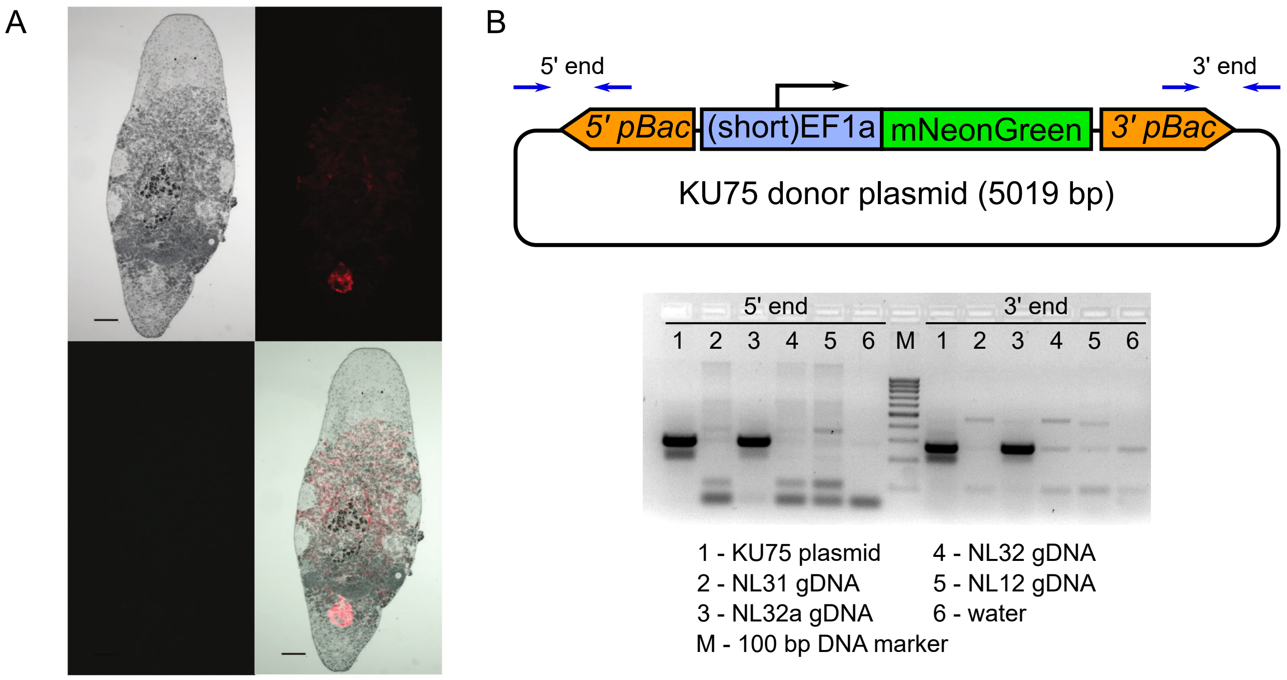

Figure S2. Distinguishing random integration from the transposon-derived transgene integration. (A) Example of the *DLG4::mScarlet-I* transgene expression pattern in *M. lignano* random integration-derived transgenic lines. Top channels from left to right: brightfield, dsRed; bottom channels: FITC, merged. Scale bar is 100 µm. (B) PCR assay for the retention of the KU75 plasmid backbone. Positions of the primers (small blue arrows) are indicated above the plasmid scheme. Orange arrow-shaped blocks correspond to the 5' and 3' *piggyBac* termini. Separation of the PCR products in a 2% agarose gel stained with EtBr is shown below the plasmid scheme. gDNA - genomic DNA. NL31, NL32 - *piggyBac*-derived transgenic lines obtained in this study. NL32a - random integration derived line. NL12 - non-transgenic wild type line. The bands were observed only for the KU75 plasmid control and the NL32a line.
