# Supplementary material for: Proof of principle for piggyBac-mediated transgenesis in the flatworm Macrostomum lignano
Source: Genetics. 2021 May 17;218(3):iyab076. doi: 10.1093/genetics/iyab076 (PMC8717057; doi:10.1093/genetics/iyab076)
Supplement: iyab076_Supplementary_Data [file iyab076_supplementary_data.zip › iyab076/GENETICS-2021-304273_Figure_S4.pdf]

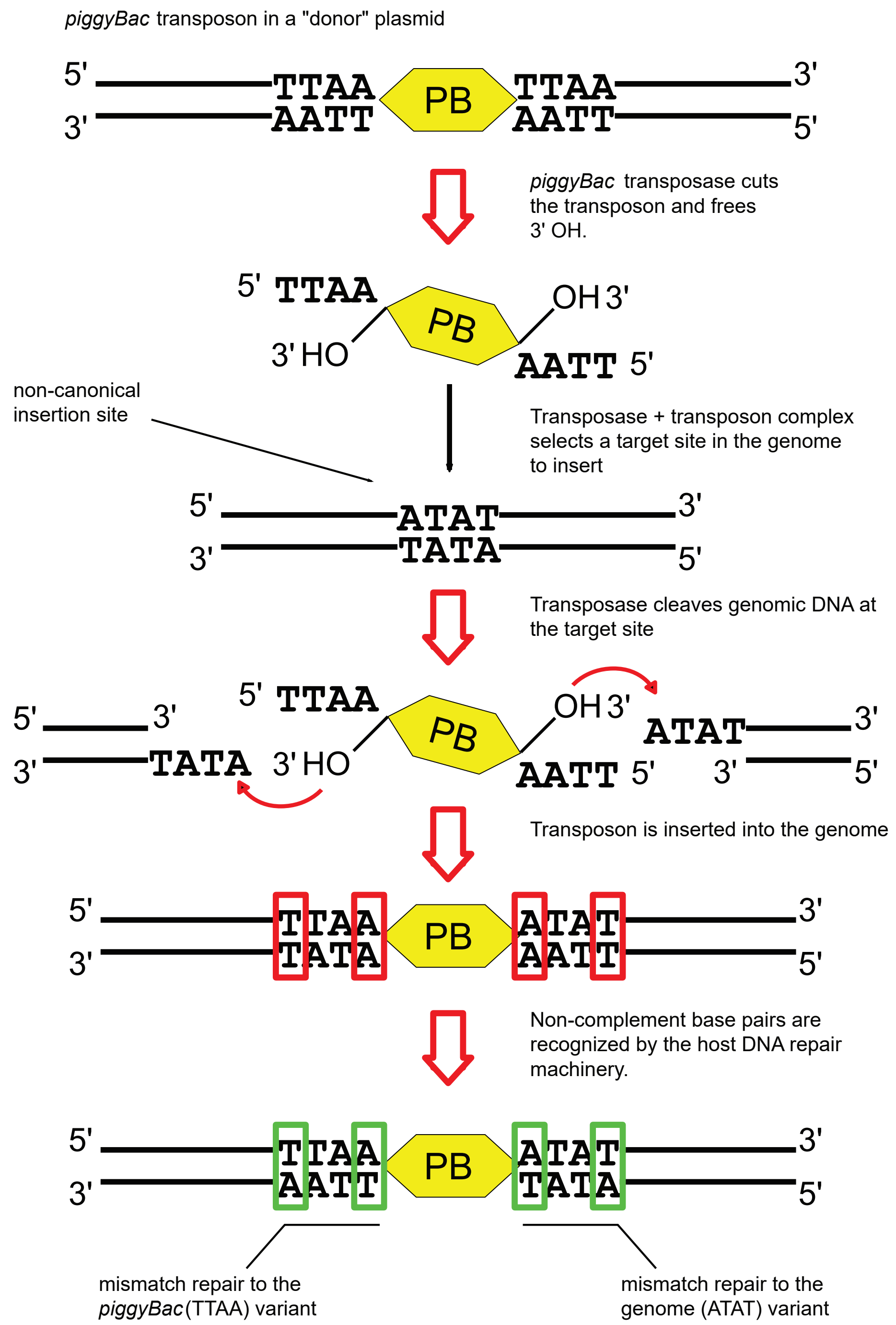

Figure S4. A hypothetical mechanism for the observed asymmetric and non-canonical target site duplication formation upon *piggyBac* (PB) insertion in the *M. lignano* genome. Based on the mechanism proposed by Li et al, 2013.
