# Supplementary material for: Proof of principle for piggyBac-mediated transgenesis in the flatworm Macrostomum lignano
Source: Genetics. 2021 May 17;218(3):iyab076. doi: 10.1093/genetics/iyab076 (PMC8717057; doi:10.1093/genetics/iyab076)
Supplement: iyab076_Supplementary_Data [file iyab076_supplementary_data.zip › iyab076/GENETICS-2021-304273_Tables_S1.pdf]

Table S1. Primers used for plasmid retention screening, inverse PCR and PST-PCR, Sanger sequencing, and genomic location validation

PCR round	*piggyBac* terminus	Primer ID	Sequence (5' — 3')
**KU75 donor plasmid retention PCR**			
-	5'	M13 long Fwd	CGACGTTGTAAAACGACGGCCAGTGAA
		piggyBac SSP R1	TTCAAAATCAGTGACACTTACCGCATTGACAAGCA
	3'	M13 long Rev	ACAGGAAACAGCTATGACCATGATTACGCCAAG
		piggyBac SSP F1	ACCTCGATATACAGACCGATAAAACACATGCGTCA
**Inverse PCR**			
1st	5'	iPB_5'-1F	AACTATAACGACCGCGTGAGT
		iPB_5'-1R	CTTACCGCATTGACAAGCACG
2nd (nested)		iPB_5'-2F	GAGTCAAAATGACGCATGATTATCT
		iPB_5'-2R	GACTGAGATGTCCTAAATGCACAG
1st	3'	iPB_3'-1F	AACCTCGATATACAGACCGAT
		iPB_3'-3R	GAGAGTCAGGAAATACTAGCAACA
2nd (nested)		iPB_3'-2F	ACAGACCGATAAAACACATGC
		iPB_3'-4R	CAAGTCTCGCAACTTTAACTTCG
**PST-PCR**			
1st	3'	piggyBac SSP F1	ACCTCGATATACAGACCGATAAAACACATGCGTCA
	5'	piggyBac SSP R1	TTCAAAATCAGTGACACTTACCGCATTGACAAGCA
2nd (nested)	3'	piggyBac SSP F2	ACAGACCGATAAAACACATGCGTCAATTTTACGCA
	5'	piggyBac SSP R2	GACTGAGATGTCCTAAATGCACAGCGACGGATT
	-	Universal tail primer	GTTGCGGCAGGTCCTCACC
1st	-	PST_*NcoI*	GTTGCGGCAGGTCCTCACCNNNNNNNNNNCCATGG
	-	PST_*PmaCI*	GTTGCGGCAGGTCCTCACCNNNNNNNNNNCACGTG
	-	PST_*SalI*	GTTGCGGCAGGTCCTCACCNNNNNNNNNNAGTACT
**Direct Sanger sequencing of PST-PCR/Inverse PCR products**			
-	5'	iPB_5'-2R	GACTGAGATGTCCTAAATGCACAG
-	3'	iPB_3'-2F	ACAGACCGATAAAACACATGC
**Genomic DNA insertion location validation (line NL30)**			
-	5'	scaf1687_PB_ins_Fwd	TCGATCTGTATTTCCAATGGCCACCTTCAAGGA
		piggyBac SSP R1	TTCAAAATCAGTGACACTTACCGCATTGACAAGCA
	3'	scaf1687_PB_ins_Rev	CAGATATATCTATAGACATGTAGCTACGGATCACTGGTAG
		piggyBac SSP F1	ACCTCGATATACAGACCGATAAAACACATGCGTCA
**Genomic DNA insertion location validation (line NL31)**			
-	5'	scaf224_PB_ins_Fwd	TTTGTCTTCCAGATCGTCCAGGTCGATG
		piggyBac SSP R1	TTCAAAATCAGTGACACTTACCGCATTGACAAGCA
	3'	scaf224_PB_ins_Rev	GCACCGTGTTTATGGATATGAATGACGATCAG
		piggyBac SSP F1	ACCTCGATATACAGACCGATAAAACACATGCGTCA
**Genomic DNA insertion location validation (line NL32)**			
-	5'	scaf808_PB_ins_Fwd	GCAAATCAGCGGACGAATAGTAGTGCATAGGT
		piggyBac SSP R1	TTCAAAATCAGTGACACTTACCGCATTGACAAGCA
	3'	scaf808_PB_ins_Rev	AATGAGGAGGTATTGGATGGCGAGCCT
		piggyBac SSP F1	ACCTCGATATACAGACCGATAAAACACATGCGTCA
