# Supplementary material for: Proof of principle for piggyBac-mediated transgenesis in the flatworm Macrostomum lignano
Source: Genetics. 2021 May 17;218(3):iyab076. doi: 10.1093/genetics/iyab076 (PMC8717057; doi:10.1093/genetics/iyab076)
Supplement: iyab076_Supplementary_Data [file iyab076_supplementary_data.zip › iyab076/GENETICS-2021-304273_Supplemental_Material_Legends.docx]

**Table S1.** Primers used for plasmid retention screening, inverse PCR and PST-PCR, Sanger sequencing, and genomic location validation.

**Figure S1.** *PiggyBac* donor and helper plasmid maps, primers, promoters and codon-optimized sequences used for creating transgenic constructs.

**Figure S2.** Distinguishing random integration from the transposon-derived transgene integration. (A) Example of the *DLG4::mScarlet-I* transgene expression pattern in *M. lignano* random integration-derived transgenic lines. Top channels from left to right: brightfield, dsRed; bottom channels: FITC, merged. Scale bar is 100 μm. (B) PCR assay for the retention of the KU75 plasmid backbone. Positions of the primers (small blue arrows) are indicated above the plasmid scheme. Orange arrow-shaped blocks correspond to the 5' and 3' *piggyBac* termini. Separation of the PCR products in a 2% agarose gel stained with EtBr is shown below the plasmid scheme. gDNA - genomic DNA. NL31, NL32 - *piggyBac*-derived transgenic lines obtained in this study. NL32a - random integration derived line. NL12 - non-transgenic wild type line. The bands were observed only for the KU75 plasmid control and the NL32a line.

**Figure S3.** Validation of *piggyBac*-derived transgene insertions by PCR using *M. lignano* genomic DNA specific primers. NL30-NL32 - *piggyBac*-derived transgenic lines obtained in this study. NL12 - non-transgenic wild type line. Names and sequences of the used primer pairs are in Table S1. (A) Schematics of the PCR reactions and expected length of the PCR products corresponding to mapped *piggyBac* locations as in Figure 2. (B) Separation of PCR products in a 1% agarose gel stained with EtBr is shown. Primers used for the PCR reaction in the lanes 1–6 were the same for the lanes 7–12. Lane M - marker DNA (Step 100 Long, Biolabmix, Novosibirsk); Lanes 1, 3, and 5 - mapping of the 5' *piggyBac* terminus. Lanes 2, 4, and 6 - mapping of the 3' *piggyBac* terminus. Sizes of the bands marked with asterisks do not exactly correspond to the expected values due to extended tandem repeat areas flanking the transposon insertion sites, which might be incorrectly assembled in the Mlig_3_7 genome assembly.

**Figure S4.** A hypothetical mechanism for the observed asymmetric and non-canonical target site duplication formation upon *piggyBac* (PB) insertion in the *M. lignano* genome. Based on the mechanism proposed by Li et al, 2013 (Li *et al.* 2013).
